# Humbugiana

**Published:** 1856-01

**Authors:** 


### HUMBUGIANA.

BY ONE WHO HAS BEEN "SOLD."

Mr. Editor.—It has been said that miracles will never cease. Whether they will or not, I cannot say; but of one thing I am certain—that the days of *Humbug* will never end. And so long as intelligent men are willing to pay for being cheated, just so long will there be the cry of Humbug.

Ever and anon, in perusing our public journals, our vision is almost blinded with flaming advertisements of *Great Discovery*. Dr. A——, who has been experimenting for over three years, expending a fortune and valuable time, has at last made the wonderful discovery which will entirely do away with all of former discoveries, fixtures and appliances; give satisfaction beyond a cavil to the fortunate patient, and make the operator independently rich. And, to allay the fears of the incredulous, he has *certificates* from many eminent Doctors, Lawyers and Divines—that it is all the author claims for it.

All this meets our eyes at one glance, before the public are aware of the existence of Dr. A——, or his wonderful discovery; without our even dreaming of the good fortune in store for us.

The above reflections are made from seeing a few days ago in one of our leading journals, the card of Dr. A——.

It appears that the Doctor has discovered that the eyes of our profession have grown weak from the constant habit of plastering them with *Patent-right nostrums ;* and, like a true philanthropist, he has set himself to work to discover a remedy. And after unwearied exertions and experiments for two or three years, he has at last produced his "*Gutta Plaster*," which he assures us will entirely remove the film from our eyes, so that we may see without the aid of patent-right spectacles.

But lest some incredulous one may doubt a perfect and *permanent* cure, he urges us to at least have confidence in it as a *temporary* substitute.

To avoid too many calling at one time on him, the Dr. has appointed agents, who will give you instructions how to apply the "Plaster," and for the moderate sum of $40 or $50, insure you a single office-right to use it.

And lest the profession should make some mistake in preparing the *plaster*, the Dr. has taken the wise precaution not to tell them how it is prepared. But instead, he has invested the sole right of preparing and vending in the firm of Smith and Brown, of Philadelphia, who will supply the profession with all they desire, at the exceedingly low price of $40 per pound.

At the same time he informs us that he has been fortunate in securing all the materials in this country fit to use in making his famous "Plaster." But lest the supply fails, and the profession become blind again, he has ordered expressly from Africa a large amount of the valuable material.

Now, brothers in the profession, come up manfully and give Dr. A——'s plaster a trial. It will not cost you much; and if you do not find it all the Dr. represents, you may at least receive *temporary* relief, until some other fortunate one may make some further discoveries, when you can "try again."

The people at large think that we make our money easily, and that we ought, for *their* especial benefit, to purchase—no matter at what price—every nostrum which Mr. Duplicity may see fit to urge upon us. No matter what past experience and failures have taught us, we are expected, by the bare word of the nostrum vendor, or one illustration of the peculiar virtues, to lend our names to certificates or puffs; to urge upon the profession the "*specific*," and to assist the vendor to fill his pockets with the hard earned dimes of his duped patrons. If, after a few months, we see where we have been "sold," we keep still lest some one accuses us of paying "too dear for the whistle." Then we remain quiet and see our neighbors "sold" or secretly try our hand again in some other similar speculation.

It is an admirable trait in our profession, that even while they are smarting and writhing under the effects of Patent rights and nostrums, they will lend their names and influence to introduce the great and wonderful, but secret remedy or fixture.

It is not the profession in the city who suffer most from the *Inventive Quack*, but those remote and back in small country places. Some of whom have never seen the city, who depend upon our journals for the improvements in the profession, see a flaming advertisement of some *new discovery*, and having a desire to keep up as well as possible in the race, scrape together every dollar they can raise to purchase the *right*. And when they have purchased it, the difficulties of using, or, as is often the case, the difficulty of procuring the apparatus or materials, makes it entirely useless on their hands. But what is more frequently the case, and still worse, after using awhile and testing the true value of their purchase, they find too late that they have purchased a useless article, and are minus their *cash*.

Thus all comes from placing too much confidence in the honesty of the vendor, who generally has but one object in view, namely, to make all the money he can out of his invention. It is not that I would under-rate the value of invention, or take away one laurel from the inventor. Far from it. I would simply caution the "uninitiated" not to be in too great a hurry to buy and pay exorbitant prices for the *statement* of the inventor before his *invention* has been tested. And at the same time I would hold up to view the false positions that the professional man places himself in, when he lends his name to every man who sees fit to offer a patent-right or nostrum to the profession.

He should remember that his name carries an influence that is oftentimes seriously felt, and one that, if the invention proves bad, may do irreparable injury to the honest victim. If it were the rich alone who were to be duped, it would not matter so much; but it is generally the class who have small means, and those who are ready to embrace every opportunity to make their fortunes. They are the ones who are the most easily injured, and often ruined.

Those men who wish to peddle off their wares to the profession, are the very ones who wish the highest respect from them, and wish to be called very liberal, while at the same moment they are sapping out the very life-blood of the profession.

They claim that it is honest, because the law protects them in it. True, but a transaction may be *honest* and still not *honorable*. Let the profession examine this subject, and see if such a course is best to elevate our profession to that standard that we are all so strenuous in contending for. Let them act as well as talk, and methinks the change will be more apparent: good may yet come out of Nazareth.

And instead of humbugging the profession with mystery and patent-rights, if each one would do what he may to enlighten others, our profession would soon be truly ranked as one of the most honorable. Let the motto on our banner be "Excelsior !" Higher and still higher—onward to perfection. But down with Quackery.

—

Our Correspondent "*who has been sold*" feels rather sore under the operation—we wish we could get him out of his difficulty—but editors are like all the rest of mankind, and after all, only have our individual opinion of matters and things.

We have heretofore expressed our opinion of the unprofessionality of patents and especially of secret remedies in our profession—regarding the latter as more unprofessional than the former, for while the patentee forbids the use of his article except on conditions, he makes the whole public, and we have his improvement as a basis for still further improvements, and this, so long as our science is progressive, is something. He however, who keeps a secret, not only forbids the use of his article or remedy except on conditions, but he keeps the whole profession ignorant of the application of those principles which he considers of such incalculable service. Both of these however, have their legal rights, and however much we might desire to have them relinquish these rights for the good of the profession, yet we see no way to force this condition of things.

Improvements which are really of utility will be used even if they are patented. Should we indeed refuse their use on this account? We think not, however much we might dislike to purchase the right; for the benefit of our patient as well as our own, pecuniarily may demand this. Because therefore an article is patented, we do not feel it our duty to withhold its true merits: hence we usually speak of such things without any reference to the trammels thrown around their use. This has been our course, while we always hold our pages open to the profession for their views not only on the merits of such articles, but also as it regards the propriety of such being patented.

The patentee must and should expect that this subject will be discussed, and often too in terms not very much to his taste; yet there is no need but this should be done in a kind, candid and courteous manner.

The fact is, we regard the patenteee not necessarily a quack; he occupies very different ground. True he *may be*

*a quack*, and so may the most scientific man; the man of secret remedies and vast pretensions is emphatically a quack. The patentee does not deal in secret remedies, and he may not have other than *just* pretensions.

There has been a great deal said about the similarity of patent and copyrights. We believe that the object of the patent law is to throw around the inventor the same protection as that given by the copyright to the author. Our correspondent has alluded to gutta percha. Well, suppose that Dr. A. had patented the process of coloring this material for dental purposes, and then thrown it into the market for general use, would not the supply and demand fix the price, just as much as it does every book that is published? This is the law of trade, and the price would soon be regulated in accordance with it. This would be the exercise of the patent principle to which few would object, and for which we alone have ever contended, and its legitimate application in this way comes as near a copyright as the law can make it.

A certain mode of preparing gold for plugging teeth may be patented, and yet who complains. The profession may all get it, and the demand will fix the quantity manufactured, and to some extent the price. The manufacturer cannot charge more for it than the best of foil is worth.

We are aware it may be said, if you allow the patenting of these materials, you must allow then the mode of using them also to be patented. We answer not necessarily, the one is no more the practice of dentistry, than the making of a forcep.

The manufacture of the material belongs to the mechanic arts, the use of the material to dental surgery.

Well; but says one, is not mechanical dentistry an art? very true, but if the dental surgeon as well as the general surgeon, finds it necessary to make use of mechanical means in the practice of his profession, need he be the less liberal and ever the less ready to sacrifice some of his legal rights on the altar of his profession.

It will be seen by Dr. Slayton's communication in this No. of the Register, that he has declined taking out a patent for his method of working gutta percha, but has simply filed a caveat for his manner of refining and coloring gutta percha. We hope he may soon see proper to give his method to the profession.

As it regards the merits of the article itself, we think there can be no doubt. We believe it is all that, it is claimed to be, and we think that Dr. Slayton has been very candid in all his statements in relation to the use of the article.

The mode of using it is very simple, and yet we believe that but few would make good, or neat work with it, without some special instruction or seeing the manipulations gone through with.

A correct plaster model of the jaw is procured as for any other work. A sheet of the gutta percha is then softened in hot water, and neatly and accurately fitted to the cast. After it has cooled, this is cut to about the size the base is desired. Then with this fastened on the plaster model, metallic dies are secured, just as for gold plates. With these a narrow silver plate is struck, up to which the teeth are soldered fast, just as they may be wanted to set in the mouth. After this is filed down, so as to be little if any wider than the base of the teeth, it is set upon the gutta percha base, and it should be first warmed, so that it will make its impression on the gutta percha.

Small scraps of the gutta percha are now to be softened over a spirit lamp, and with the point of a bistoury pointed instrument heated over the lamp as occasion may require, these small pieces are united to the base plate, and brought up inside and outside over the silver plate, and around the necks of the teeth, just to that extent the operator may desire. With this instrument heated, it may be pressed and worked in until it becomes perfectly united, and then trimmed off to the size wanted.

For partial operations clasps may be attached, and the gutta percha so accurately fitted to the necks of the clasp teeth, that but little if any metal could come in contact with the teeth.

For such cases where the metal would have to be exposed, gold might be used instead of silver, for the plate to which the teeth are fastened.

We believe that gutta percha will be used a great deal in dental practice. In all those operations requiring gum teeth, and which must necessarily be wanted only two to four years, this kind of work can be made of great use. With it, it is so easy to make such alterations as will always insure a good fit. The gutta percha may all be removed, and with the same teeth the operation renewed in a few hours.

